# The psychosocial impact of childhood dementia on children and their parents: a systematic review

**DOI:** 10.1186/s13023-023-02859-3

**Published:** 2023-09-07

**Authors:** Suzanne M. Nevin, Brittany C. McGill, Lauren Kelada, Gail Hilton, Megan Maack, Kristina L. Elvidge, Michelle A. Farrar, Gareth Baynam, Naomi T. Katz, Leigh Donovan, Sarah Grattan, Christina Signorelli, Kaustuv Bhattacharya, Kenneth Nunn, Claire E. Wakefield

**Affiliations:** 1 School of Clinical Medicine, UNSW Medicine and Health, Discipline of Paediatrics and Child Health, Sydney, Australia; 2 Behavioral Sciences Unit, Kids Cancer Centre, Sydney Children’s Hospital, Randwick, Australia; 3 Childhood Dementia Initiative, Sydney, NSW Australia; 4 Department of Neurology, Sydney Children’s Hospital, Randwick, Australia; 5 Faculty of Health and Medical Sciences, Division of Paediatrics, University of Western Australia, Western Australia, Australia; 6 Rare Care Centre, Perth Children’s Hospital, Perth, WA Australia; 7 Telethon Kids Institute, Perth, WA Australia; 8 Victorian Paediatric Palliative Care Program, Royal Children’s Hospital, Melbourne, VIC Australia; 9 Genetic Metabolic Disorders Service, Sydney Children’s Hospitals’ Network, Randwick and Westmead, Australia; 10 Faculty of Medicine and Health, Discipline of Genomics, Sydney University, Westmead, Australia; 11 Department of Psychological Medicine, Children’s Hospital at Westmead, Sydney, NSW Australia

**Keywords:** Child, Neurodegeneration, Dementia, Psychosocial, Healthcare, Parent

## Abstract

**Background:**

Childhood dementias are a group of rare and ultra-rare paediatric conditions clinically characterised by enduring global decline in central nervous system function, associated with a progressive loss of developmentally acquired skills, quality of life and shortened life expectancy. Traditional research, service development and advocacy efforts have been fragmented due to a focus on individual disorders, or groups classified by specific mechanisms or molecular pathogenesis. There are significant knowledge and clinician skill gaps regarding the shared psychosocial impacts of childhood dementia conditions. This systematic review integrates the existing international evidence of the collective psychosocial experiences of parents of children living with dementia.

**Methods:**

We used the Preferred Reporting Items for Systematic Reviews and Meta-Analyses (PRISMA) guidelines. We systematically searched four databases to identify original, peer-reviewed research reporting on the psychosocial impacts of childhood dementia, from the parent perspective. We synthesised the data into three thematic categories: parents’ healthcare experiences, psychosocial impacts, and information and support needs.

**Results:**

Nineteen articles met review criteria, representing 1856 parents. Parents highlighted extensive difficulties connecting with an engaged clinical team and navigating their child’s rare, life-limiting, and progressive condition. Psychosocial challenges were manifold and encompassed physical, economic, social, emotional and psychological implications. Access to coordinated healthcare and community-based psychosocial supports was associated with improved parent coping, psychological resilience and reduced psychological isolation. Analysis identified a critical need to prioritize access to integrated family-centred psychosocial supports throughout distinct stages of their child’s condition trajectory.

**Conclusion:**

This review will encourage and guide the development of evidence-based and integrated psychosocial resources to optimise quality of life outcomes for of children with dementia and their families.

**Supplementary Information:**

The online version contains supplementary material available at 10.1186/s13023-023-02859-3.

## Key points

- A synthesis of the shared psychosocial experiences and impacts of conditions causing childhood dementia is warranted.
- Parents share a tranche of overlapping challenges owing to limited access to psychosocial resources for their child’s dementia condition.
- Severe neurocognitive decline is associated with higher parent psychological distress and isolation.
- Coordinated psychosocial interventions are required to improve outcomes for the family unit.
- Growing childhood dementia public awareness may activate earlier access to coordinated, sustainable and integrated supports.

**Supplementary Information:**

The online version contains supplementary material available at 10.1186/s13023-023-02859-3.

## Introduction

Childhood dementia comprises a group of devastating, predominantly neurodegenerative conditions, characterised by global and progressive neurocognitive decline, relative loss of developmental skills, as well as a shortened life expectancy in infants, children, and adolescents [1]. Whilst individually rare, surveillance studies have reported incidence rates between 10 and 60 per 100,000 births [2–4] which is on par with other well-recognised conditions such as cystic fibrosis (1 in 3139 births) [5, 6]. Noting that there are differences in population, terminology, definition and grouping of disorders in these studies. Examples of conditions that consistently fit the definition of childhood dementia include Batten disease, Sanfilippo syndrome, Niemann–Pick disease types A and C, Tay-Sachs disease, metachromatic leukodystrophy, and some mitochondrial disorders, amongst others [7–10]. While global neurocognitive decline is a core deficit, each individual childhood dementia-causing disorder is clinically heterogeneous, with variable patterns of disease progression that affects every aspect of adaptive function [9–11]. Pathways to identify causes for this clinically severe group of conditions are complex [12]. Treatment options are limited for this group of rare diseases resulting in a severely shortened life expectancy ranging from infancy (e.g. Gaucher disease type 2 and nonketotic hyperglycinemia glycine encephalopathy) [13, 14], to ~ 50 years of age (e.g. Rett syndrome)[15]. It has been estimated that 75% of children with dementia die before the age of 18 [16]. While extensive progress has been made in understanding the underlying pathophysiology for individually rare childhood dementia conditions, with a growing number of genetic causes still being discovered, scientific advancements have yet to translate into accessible healthcare services and psychosocial supports for affected families [11, 17, 18].

Often, children will demonstrate typical development, then decelerate their progress, with substantial regression and global deterioration [19]. In other cases, syndromes can be so severe that the impact on neurodevelopment and associated neurocognitive decline begins from a low baseline of development, resulting in the prevention of the attainment of developmental skills [20–22]. Although symptomatology can vary across disorder groups, children commonly develop a spectrum of co-existing and progressive symptoms related to the primary disease mechanism that impact cognitive, behavioral, and physical domains, including organ systems associated with their dementia condition [11, 23]. Childhood dementia shares several hallmark features akin to adult dementia, including: decline in cognitive ability, memory loss, wandering and restlessness, emotional difficulties (e.g. anxiety, fear, panic attacks), personality and behavioral changes (e.g. aggression, irritability, hyperactivity)[19]. Moreover, children with childhood dementias suffer severe sleep disturbances, movement disorders (e.g. muscle spasms, tremors), deterioration of communication skills, loss of vision and hearing, mood disorders, psychosis (including hallucinations and delusions) and incontinence [11]. Childhood dementia conditions are also severely life-limiting and life-threatening; causes of death include respiratory complications (e.g. pneumonia), neurological complications (e.g. intractable epilepsy), or cardiac events [16]. Throughout the course of the illness acute global brain dysfunction including delirium, encephalopathy are common and towards the end of life prolonged periods of stupor and coma [1, 7, 24].

Given the non-specific initial presenting symptoms, the rarity of the individual conditions, and associated limited natural history data, diagnosis of a childhood dementia condition is typically delayed, sometimes for years after the first symptoms appear [12, 25]. Children are commonly misdiagnosed with autism, developmental or intellectual delay [25] and families can face a ‘diagnostic odyssey’ in the search for a diagnosis for their child. Research in other rare disease groups has demonstrated that delayed and prolonged diagnoses, lack of treatment options and chronically unmet needs negatively impact parents’ physical and psychological wellbeing [26–30]. There has been an increasing focus on interdisciplinary collaboration and cross-pollination of knowledge to enhance healthcare services, including diagnostics and therapeutic advancements for individuals living with rare diseases [18, 31]. Emerging from this climate, the Childhood Dementia Initiative was launched in Australia in 2020 with the purpose to transform research, care and quality of life for children with dementia by: bringing together the many individual ‘siloed’ conditions; focusing on the commonalities of childhood dementia; fostering diverse but complementary approaches; driving collective progress.

Psychosocial research specifically considering the collective healthcare experiences and psychosocial impacts of childhood dementia conditions on parents is currently lacking. This lack of evidence represents a significant obstacle for clinicians, patients, and their families and has delayed the development of a multidisciplinary healthcare approach and evidence-based psychosocial supports [32, 33]. Psychosocial research is important to facilitate better understanding of the unique caregiving demands placed on parents and to explore how parents respond to and cope with the challenges of their child’s rare disease. This review will contribute to the knowledge base to inform coordinated healthcare and community supports and the development of evidence-based psychosocial resources to address the unmet needs of parents.

Our review focused on three research questions:

1. What are **parents’ experiences** of navigating their child’s **healthcare**?
2. What is the current evidence regarding **the psychosocial impacts** of childhood dementia, as reported from the parent perspective?
3. What are parents’ **information and support needs**?

## Method

We structured the review using the Preferred Reporting Items for Systematic Reviews and Meta-Analyses (PRISMA) guidelines [34]. We searched the PROSPERO database [35] prior to commencing this review and published a protocol for this review (CRD42021291858).

### Search strategy

We conducted the search and article selection process between February and April 2022, identifying original studies by searching four electronic databases including PubMed, PsycINFO, Embase, and the Cumulative Index to Nursing and Allied Health Literature (CINAHL). We applied a detailed and extensive review of indexing terms used in a set of articles that met the search inclusion criteria to identify suitable search terms. We then consulted clinical subject matter experts (KE and MF) to refine the set of search terms and to ensure the relevance and clinical applicability of our search. In addition, we consulted with an independent research librarian to assess our search strategy completeness. We modified individual search strategies according to the database MeSH/subject headings and employed database searching conventions for identifying the use of single and plural terms, as well as to account for different spellings of search terms to create a comprehensive search (see Additional file 1: Table S1).

We employed the following search strategy (parents OR caregiver OR family OR child) AND (childhood dementia OR mental deterioration OR progressive cognitive decline OR cognitive decline) AND (psychosocial OR quality of life OR health service needs and demands OR mental health OR coping). We also conducted a search on Google Scholar and reviewed the reference lists of included studies.

### Eligibility criteria

We determined the article eligibility criteria using the PICOS characteristics [36] (i.e., characteristics describing the study population (P), illness/condition (I), comparison condition (C), outcome (O) and study design (S)). We included original, peer-reviewed articles published in English addressing the parent-reported psychosocial impacts of caring for a child with a childhood dementia. To surmount the heterogeneity between childhood dementia conditions, we adopted the clinical criteria proposed in Box 1, adapted from published case definitions [11]. When studies included sub-groups of parents of healthy children, general population norms, or parents of children with chronic diseases, we included these sub-groups as comparisons. Qualitative, quantitative and mixed-methods studies were included if 1) they assessed the parent-reported psychosocial impacts of caring for a child with a dementia (< 18 years); and 2) > 50% of the sample were parents of children with a childhood dementia (that met the criteria according to the below text description). The inclusion criteria for childhood dementia included any child (under 18 years of age at symptom onset) with any illness that fulfilled all the following criteria:

**Box 1:** Inclusion and exclusion criteria for childhood dementia definition*

Inclusion: any child (under 18 years of age at symptom onset) which has any illness that fulfils all the following criteria:
• Multiple losses of already attained cognitive developmental skills
• Duration of illness greater than 3 months
• Skill loss most likely due to CNS dysfunction
• Evidence of generalised (not merely focal) brain dysfunction
• Has a condition which will in the future, in all probability, lead to progressive deterioration as above
Exclusion:
• Conditions associated with static intellectual losses (e.g., infectious, traumatic, or anoxic insults) (a)
• Conditions mainly associated with episodic cognitive impairment (e.g., in the context of acute metabolic crises)
• Conditions with primary cognitive decline because of epilepsy [1, 7] (b)

*Adapted from the only published case definitions identified for childhood dementia

a. Static neurocognitive decline was excluded as the label of static skill loss as dementia was not acceptable to clinicians [1]. This is the rationale for excluding any forms of static or transient cognitive decline such as in infectious or toxic encephalopathies, acute metabolic crises (phenylketonuria, urea cycle disorders), or traumatic and anoxic injuries (head trauma, drowning).

b. Whilst the epileptic encephalopathies can cause episodic cognitive decline, this is not currently seen as temporally progressive neurodegeneration [37–39].

*A note on terminology

The term childhood dementia is not ubiquitously employed as standard throughout the medical literature. Conditions that cause progressive cognitive decline in childhood have been grouped as 'progressive intellectual and neurological deterioration' (PIND) in children [7], progressive childhood encephalopathy ([8, 9] and childhood dementia [1], albeit with slightly different definitions. At present, Human Phenotype Ontology (HPO) and Online Mendelian Inheritance in Man (OMIM) do not catalogue search terms to identify childhood dementia conditions. Mental retardation and dementia are terms that are far broader than childhood dementia. 'Mental retardation' includes conditions where IQ is low, but IQ does not necessarily decline as it does in childhood dementia. 'Dementia' includes adult-onset conditions such as Alzheimer's disease. This inconsistency of both language and definition is hampering efforts to understand this group of diseases and very little is known about the shared needs of families. This was our reasoning to conduct this systematic review, to provide the evidence needed to advocate for improved care and treatment of this group of patients. Concomitant interdisciplinary research and advocacy efforts are currently underway to improve consistency of definition, terminology and coding for this group of diseases [40].

We excluded: case studies, unpublished dissertations, clinical intervention trials, validation studies of quality of life instruments, and studies that did not directly report on parents’ self-reported psychosocial impacts of caregiving. We also excluded articles which reported on the aggregate findings of different disease groups, wherein the specific impact of childhood dementia conditions on parents could not be ascertained.

### Selection criteria

**Fig. 1:**
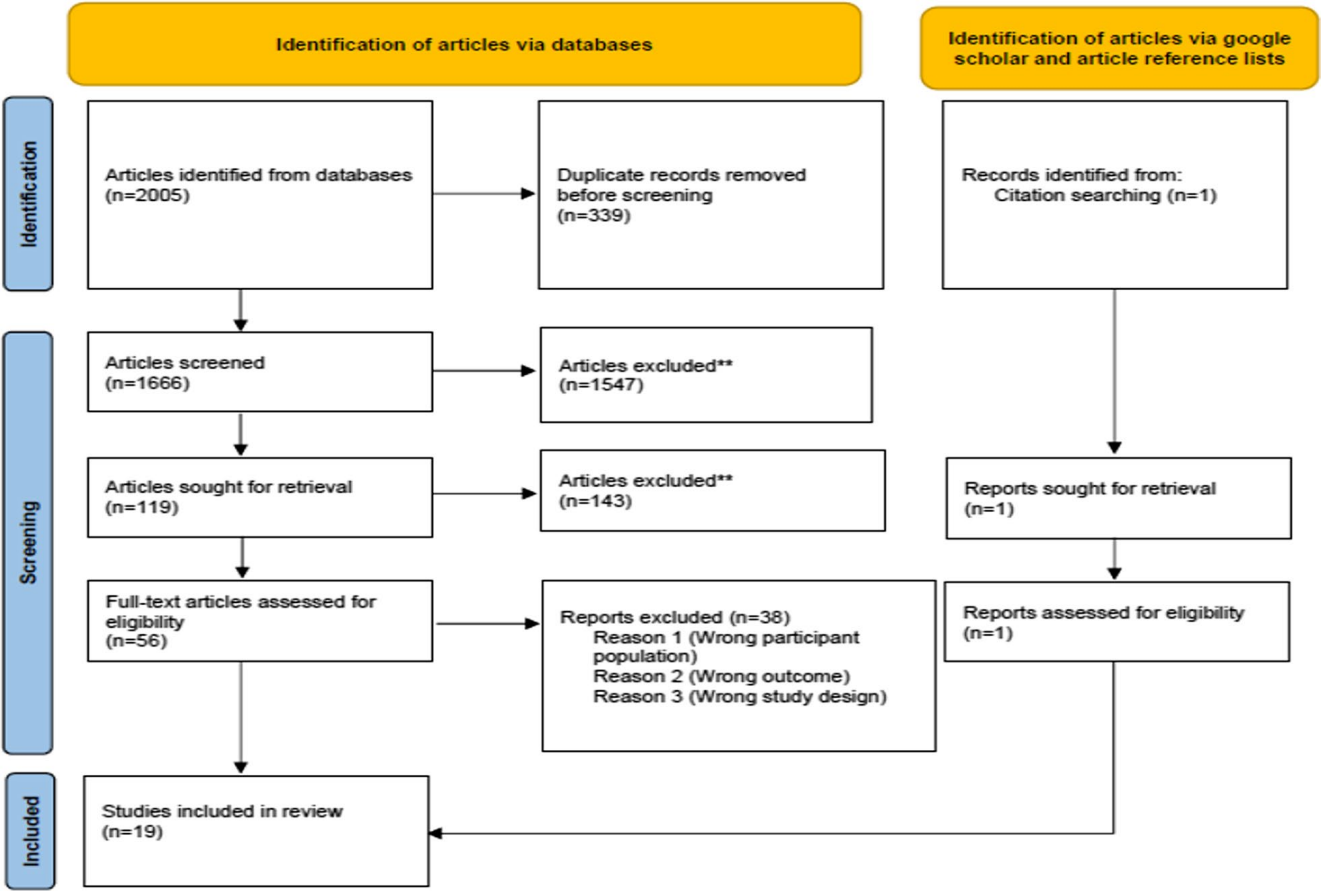
PRISMA diagram

We exported all citations from our individual database searches using Endnote X7 (Thomas Reuters) and removed duplicates. We imported and merged all articles in RAYYAN, which is a specialized software program for conducting systematic reviews [41]. SMN and BM independently screened the titles and abstracts of the studies which were potentially eligible for inclusion using RAYYAN QCRI, to identify citations that related to the psychosocial experiences of parents and families of children with childhood dementia. To ensure agreement and equal understanding of the eligibility criteria between the two reviewers, a pre-test of the criteria was conducted, with each reviewer screening 100 abstracts and comparing their decisions before they continued the screening. SMN and BM independently reviewed all full texts, noting their decisions for including or excluding articles in the RAYYAN system. Wherever uncertainty regarding clinical diagnostic criteria for childhood dementia was arose, SMN consulted a clinical expert in childhood dementia (KE) to make a final decision. Figure 1 shows the PRISMA flow diagram of the identified and selected citations [42].

### Data extraction

SMN lead the data extraction for all 19 studies and BM independently extracted data for 20% of studies (randomly selected) to monitor accuracy of the process. Information extracted included [1] study characteristics (author, year and country of publication, study design); [2] parent characteristics (gender, age range) and patient characteristics (childhood dementia diagnosis, gender, age range of child); [3] recruitment methods and outcome assessment methods (questionnaire, interview, focus group); [4] relevant thematic findings (healthcare experiences, psychosocial impacts, information and support needs). Summary and evidence tables were created for this purpose.

### Quality analysis

We used the QualSyst tool [43] to assess the research methodology quality and risk of bias of included studies. SMN and BM rated the studies according to either qualitative or quantitative criteria. To ensure fairness in ratings, if the studies included both qualitative and quantitative elements but were not explicitly stated to be mixed methods, then SMN and BM evaluated the primary research design described in the methods. SMN and BM rated the studies independently, compared assessments, and resolved any discrepancies through discussion until they reached consensus.

### Methodological Rigour

The QualSyst scoring system contains 14 items to assess quantitative studies, with each item scored as 0 (no), 1 (partial), 2 (yes), or ‘not applicable’. The same ranking process is used to assess qualitative studies that are rated on a scale containing 10 items. Total summary scores were obtained for each study (see Additional file 1: Tables S2 and S3) and converted to a quality index score, ranging 0–1. To be included in the review, studies required a quality index score of 0.5 or above [43]. This threshold was chosen following consideration of the quality score distribution and the relatively small number of articles that met our review inclusion criteria (Additional file 1: Tables S2 and S3). To account for the heterogeneity of included articles and outcomes reported, we performed a content analysis [44] and narrative synthesis of the results to characterize the impact of childhood dementia conditions on parents’ healthcare experiences and psychosocial outcomes [45]. This included exploring and comparing patterns of thematic findings highlighting the effects parents’ healthcare experiences and factors shaping parents’ psychosocial impacts across the included studies [46]. We also considered the quality of the methodologies, to guide clinical practice recommendations and future research directions.

## Results

**Fig. 2:**
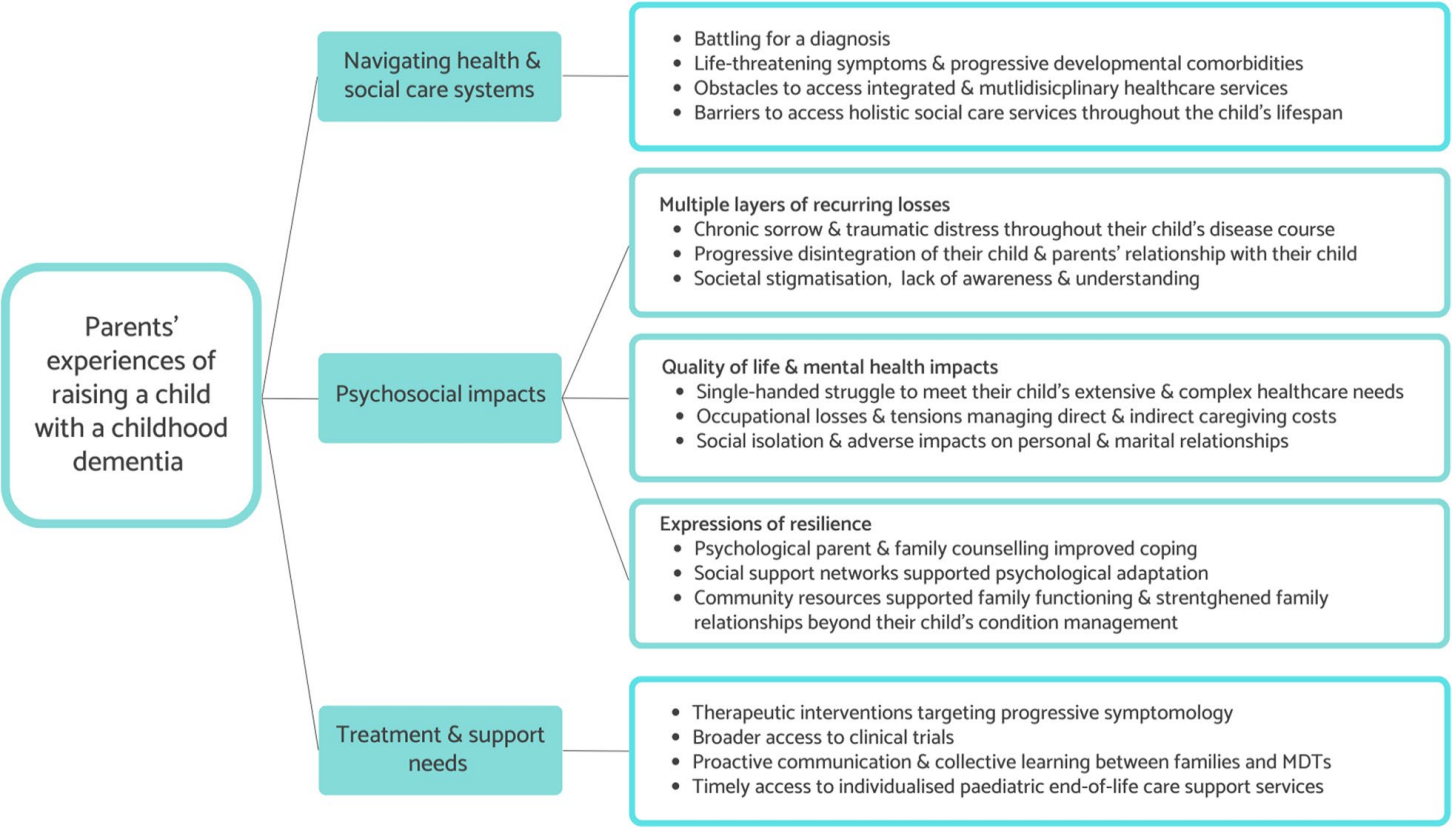
Thematic categories of parents experiences

We identified 19 eligible articles, including 7 qualitative [47–53] and 12 quantitative studies [54–65] representing the perspectives of 1856 parents of children across 21 different dementia-causing conditions. The mean quality score for the quantitative studies was 0.91 and the mean quality core of the qualitative studies was 0.94 (see Additional file 1: Table S1). We excluded one article that did not provide sufficient detail of the study methodological rigour to be included in this review. These studies stemmed from 11 countries: the USA [48, 50, 52, 55, 61, 63, 64], Canada [47, 57], Germany [58, 62], Australia [65], Ireland [49], Japan [60], Poland [54], Spain [53], Sweden [51], the Netherlands [59], and the United Kingdom [56]. Several studies ascertained data from international populations using online platforms (e.g. rare disease advocacy websites) to maximise recruitment. Of the 19 studies, 15 were cross-sectional [47, 48, 50–52, 54–62, 64], 3 were longitudinal [49, 63, 65], and one study comprised a multi-perspective cohort [53]. Table 1 lists the dementia causing conditions examined in each included study. Sixteen studies examined the perspectives of both mothers and fathers [47, 48, 50–53, 56–60, 62–66], three studies did not specify parents’ gender [49, 54, 55] and one study included only mothers [61]. There was large variance in sample size within the included studies ranging from N = 8 [51] to N = 727 participants [63]. Twelve included more than 20 participants [48, 50, 52–54, 56–62], while four studies included more than 50 participants [55, 63–65]. In line with our review questions, we extracted the results from the articles into three thematic categories focusing on [1] parents’ healthcare experiences; [2] psychosocial impacts; and [3] information and support needs (Fig. 2). A summary of the results of each study can be found in Tables 1 and 2.

**Table 1:** Qualitative and quantitative articles included in this review

Authors	Year	Country	Research design	Study aim/purpose	Recruitment methods	No. of participants (% male, female)	Parent age range/mean age	Child age range/mean age	Patient diagnosis	Data collection methods
Hoffmann et al. [58]	2021	Germany	Cross-sectional	To offer parents specific effective day-today coping measures:	In cooperation with the German MPS Society, a convenience sample of all registered families of children with MPS I, -II & -III & neurological involvement (n = 268) were invited to participate by mail in the summer of 2017	N = 37 (4 males & 33 females)	children were male in 52.9% (n = 18)	M = 8.7 (SD = 4.23) years of age	MPS I, -II or -III	Semi-structured questionnaires. Impact on interfamilial relationships used two-sided VAS-scales (range − 2.50 to + 2.50). Advice for ‘recently diagnosed’ families was collected through free text investigation
Yazdani et al. [47]	2022	Canada	Cross-sectional	To explore the extent to which parents think modifications of healthcare services have affected their families	Parents were identified through the Leukodystrophies & Neurometabolic Disorders Clinic at the Montreal Children’s Hospital of the McGill University Health Centre. Sampling was purposeful, & not r&om. Eligible parents were contacted until data saturation was reached	N = 13 (4 males & 9 females)	36–54 years	3–23 years	Genetically Determined Leukoencephalopathies	Semi-structured interviews & sociodemographic form
Eichler et al. [50]	2016	USA	Cross-sectional	To identify relevant clinical/quality-of-life outcomes for patients/parents	Participants were identified via patient support groups: in the United States, France, Germany & Colombia	N = 23 (14 males & 9 females)			Metachromatic leukodystrophies	Semi-structured qualitative interviews & 1 focus group
Needham et al. [55]	2014	USA	Cross-sectional	To describe a population of patients with MPS II in the domain of adaptive functioning, & to examine the impact of MPS II on family functioning & relationships	Patients were recruited through national MPS societies in English-speaking countries, the hunterpatients.com website, the MPSforum.com website, & through contacting geneticists in the United States	N = 73	not reported	12.52 years (SD = 8.88). all patients were male	MPS II (Hunter syndrome)	Questionnaire including stadardised measures (Vinel&-II & PedsQL Family Impact Module) & purpose designed measures (demographics % supplementary Qs for qualitative analysis)
Krantz et al. [51]	2021	Sweden	Cross-sectional	To investigate parents' experience of having a child with CLN3 & how these experiences can be related to family resilience	Parents of children diagnosed with CLN3 disease attending or who had attended Queen Silvia's Hospital in Gothenburg, Sweden were contacted by the research team in February/March 2021	N = 8 (3 males aged 40–51 years & 5 females aged 41–51)		11–24 years, (N = 5 & one patient deceased)	CLN3 disease	Semi-structured interviews
Porter et al. [52]	2021	USA	Cross-sectional	To explore parent-reported impact of Sanfilippo syndrome symptoms on the patient & family, which symptoms were most important to be addressed by future treatments, & what degree of treatment benefit would be considered meaningful	Cure Sanfilippo Foundation recruited participants using email & social media, through the ConnectMPS Registry communication, & by snowball recruitment through existing formal informal parent support networks	N = 25 (6 males & 19 females)	29 to 65 years, with a median age of 38 years	4–36 years with a median age of 8yrs	MPSIII subtype A (17), subtype B (6), or subtype C (2)	Focus groups (× 3) & a questionnaire
Grant et al. [56]	2013	UK	Cross-sectional	To examine the psychological impact of parenting a child with MPS III using valid & reliable measures, with a control group of parents of children with ID, to examine syndrome specific effects of parenting a child with MPS III	Families with a child with MPS III were recruited from the UK MPS Society & the outpatient clinic of Metabolic Medicine at Manchester, as part of a large-scale investigation into the sleep & circadian rhythms of children with MPS III	N = 23 (7 males & 16 females) (& 23 parents of children with ID; 3 M & 20 F)	M = 35.8 (23–49 years)	M = 8.63 (2–15 years)	MPS III	The learning disability casemix scale, ECBI, RSA, MSPSS, Brief COPE, PIP, GHQ-12
Somanadhan et al. [49]	2016	Irel&	Longitudinal	To underst& parents’ experience of living & caring for a child with MPS (1). To examine the knowledge & underst&ing of MPS from the perspective of parents (2). To explore the impact of regular hospitalisation of children living with MPS on family life (3)	Families were recruited from the outpatient clinic of the National Centre for Inherited Metabolic Disorders (NCIMD), Irel& where access to this specialist cohort group was more readily available	N = 8	not collected	6 months-22 years	MPS I Hurler syndrome, MPS II (Hunter syndrome), MPS III (Sanfilippo syndrome)	Interviews
Kuratsubo et al. [60]	2008	Japan	Cross-sectional	To assess mental health in parents of patients with the childhood cerebral form of X-linked adrenoleukodystrophy (CCALD) & to investigate factors relating to psychological problems in order to improve clinical management & quality of life	Mental health examinations & a questionnaire were sent to 40 ALD families who were members of the Japanese Association of ALD families	N = 37 parents (43% males)	M = 47 years (32–60)	M = 16.8 years (8–24), Years from disease onset 8.7 years (2–16)	X-linked adrenoleukodystrophy (CCALD)	Questionnaire & psychological examinations (BDI-II, GHQ60, STAI)
Varvogli et al. [61]	1999	USA	Cross-sectional	To investigate the psychological profile of mothers whose children carry a mitochondrial disorder	Participants were mothers of children followed by the Mitochondrial Clinic at Children’s Hospital	N = 42	M = 35 (range 23–49 years)	mean age 6 years (N = 50) (range 8 months-22 years)	MELAS, Kearns-Sayre syndrome, PDH deficient, NARP syndrome, Cytochrome oxidase deficiency, Complex I deficiency, Complex III deficiency, Leigh disease, Barth syndrome, Multiple complex deficiency	Phone interview & multiple-choice questionnaire (MMPI-2) to assess personality & psychopathology
Conjin et al.	2018	Netherl&s	Cross-sectional	To assess depression, & parental distress, as well as posttraumatic stress symptoms, in mothers & fathers of MPS III patients compared to Dutch reference groups	Parents of all living MPS III patients under care at the Academic Medical Center (AMC), Amsterdam, were invited by letter to participate in this cross-sectional study Parents who gave permission to participate received an e-mail with a personal link to online questionnaires	N = 57 (19 males & 26 females)	M = 20, age range 5–38 years		MPS III	scales (HADS, DT-P, SRS-PTSD)
Dermer et al. [57]	2020	Canada	Cross-sectional	To characterize stress experienced by parents of children affected by genetically determined leukoencephalopathies, along with demographic & clinical factors correlating with higher parental stress & demonstrate the feasibility of the Parenting Stress Index–4th Edition	Parents were eligible to complete the questionnaire if their child was between 1 month & 12 years of age. All patients were followed at the Montreal Children’s Hospital of the McGill University Health Center in Montreal, Quebec, Canada. Patients were included if they had a molecular diagnosis of genetically determined leukoencephalopathy or evidence of genetically determined leukoencephalopathy based on the brain MRI pattern	N = 55 (21 males & 34 females)		Between 1 & 12 months old (11%), 1–4 years (33%), 5–7 years (17%), 8–12 years (39%)	Genetically Determined Leukoencephalopathies	PSI-4 (3 domains of parenting stress), Electronic & paper charts were reviewed for each patient & clinical features were recorded & Clinical notes were also used to evaluate the degree of the child’s disability, using validated measures
Ammann-Schnell et al. [62]	2021	Germany	Cross-sectional	To investigate the impact of these diseases on the affected families, e.g. mothers, fathers & non-affected siblings	Families recruited within the nationwide Leukonet natural history study, patient support groups in Germany [ELAGermany, MLD support group “Weisse Wolke” & PCH- Familie e.V.])	N = 43 families, (35 males & 41 females)		11.6	MLD = 30	Semi-structured questionnaire, consisting of a general part & the Pediatric Quality of Life Inventory (PedsQL™) Version 2.0 Family Impact Module (FIM)
Bose et al. [48]	2019	USA	Cross-sectional	To characterize the ZSD caregiver emotional experience in order to develop a comprehensive picture of the specific influences & interactions within a caregiver's daily life	All participants for this study were members of the Global Foundation for Peroxisomal Disorders (GFPD; https://www.thegfpd.org/)., study recruitment fliers were posted on the GFPD website & Facebook page to solicit enrolment. Individuals self-selected to participate	N = 37 (12 males, 25 females)	25–64 years		30 children with ZSD & 7 children with D-bifunctional protein deficiency (DBPD), a clinically-similar peroxisome disorder, participated in the study	
Killian et al. [63]	2022	USA	Longitudinal	To characterize relationships between child & caretaker characteristics & caretaker quality of life		N = 727 & N = 220 (30%) had 5 years of follow-up	M = 38 years	M = 9	Rett syndrome	Semi-structured focus group(s)(× 3) & demographic questionnaire
Rozensztrauch et al. [54]	2021	Pol&	Cross-sectional	To underst& the clinical presentation of RTT in relation to QoL & family functioning	Eligible parents were contacted & mailed traditional pencil-&-paper questionnaires together with a stamped, self-addressed envelope in which they were to return the completed survey to the research team. The invitation to participate in the study included a letter & an information packet explaining the study & the questionnaires to complete	N = 23		M = 7 (2–12 years)	Rett syndrome	An author-designed questionnaire (ADQ), the Pediatric Quality of Life Inventory 4.0 generic core scales; PedsQL 4.0), & the PedsQL Family Impact Module (PedsQL-FIM)
Lamb et al. [64]	2016	USA	Cross-sectional	To investigate factors related to family functioning & adaptation in caregivers of individuals with Rett syndrome (RS)	Participants were recruited from 4 clinics across the eastern & south-eastern United States, the 25th Annual Education & Awareness Conference for Rett Syndrome, & various RS organizations & websites. All interested individuals were invited to complete the paper surveys that were mailed to their homes or to visit the online version available on a SurveyMonkey1 platform	N = 396 (90% females)	M = 43, range 22–74 years	M = 13, range 1-50yrs 98% females	Rett syndrome	Quantitative survey (available paper & electronic form) with validated measures of parental self-efficacy, coping methods, family functioning, & adaptation
Palacios-Ceña et al. [27]	2019	Spain	Multi-perspective cohort study	To explore the experiences of a group of caregivers of children with Rett syndrome with regards to living & caring for their children	The researchers explained the purpose & design of the study to the individuals who met the inclusion criteria during an initial face-to-face contact session. A one-week period was then allowed for patients to decide whether or not they wished to participate & they were given a copy of the informed consent for them to review. In a second face-to-face session, they were asked to provide written informed consent & permission to tape the interviews	N = 31; (14 males, 17 females)	M = 45	M = 12 years, range 3–38 years	Rett syndrome	In-depth interviews, focus groups, researchers’ field notes & caregivers’ personal documents
Mori et al. [65]	2019	Australia	Longitudinal	to examine the trajectory of parental well-being among Australian families caring for a child with Rett syndrome over a period of 9 years	Data were sourced from the Australian Rett Syndrome Database (ARSD), housed at the Telethon Kids Institute in Perth, Western Australia.. Data on a comprehensive range of family & child characteristics have been collected via family questionnaires in 1996, 2000, 2002, 2004, 2006, 2009 & 2011, developed in accordance with the specific aims of each wave of data collection in collaboration with a family reference group	N = 198; (92% females)	M = BL: 41 (27-64yrs range) to M = 48 years at end timepoint (30-72trs range)		Rett syndrome	Purpose designed questionnaires including McMaster Family Assessment Device (FAD) to assess family functioning & parental wellbeing using the short Form 12 Health Survey (SF-12)

**Table 2:** Qualitative and quantitative articles included in this review

Authors	(1) Healthcare experiences with their child?	(2) Psychosocial impacts of childhood dementia	(3) Parents’ information & support needs
Hoffmann et al. [58]	MPS I patients reported lower frequency of challenging behaviour & better perceived manageability than MPS II & -III Aggression, hyperactivity, agitation, orality, & sleep disturbance highly frequent & stressful symptoms Medication & non-medication approaches rated similarly effective	Parents grow into a state of acceptance, largely uninfluenced by child behavior or state of illness Social exchange & private space are important to strengthen personal & interfamilial functioning	Support managing challenging & uncooperative behaviors Family networking & sharing of management strategies
Yazdani et al. [47]	Perceived impact of the pandemic on healthcare services: Delays & losses: cancelled appointments, prolonged waiting time for services, loss of home care support, loss of school-related services, parental decision to delay services for the safety of their family Management of child’s needs independently associated with perceived amplification of child’s symptoms	Advantages: reduced emotional & physical burden of going to the hospital; reduced travel time; more quality time with loved ones; saved costs associated with gas & parking; reduced work absenteeism; reduced child & parent exhaustion; improved communication between parents & healthcare professionals Concern: parents experience a higher level of stress owing to the shortage of services & the child’s vulnerability Stress fear that physicians might miss important clinical features regarding child’s regression	Desire for telemedicine in their child’s long-term care Telemedicine only employed when physical examination is not necessary, when the child is stable & well known to the physician Preference of video consultations over phone appointments
Eichler et al. [50]	Diagnostic process: misdiagnoses, difficulty with length of time taken to obtain a final MLD after symptom onset Most troublesome symptoms: immobility & respiratory difficulties Frequent healthcare visits: 8/22 patients experienced 11 hospitalizations since diagnosis, & 14/22 parents reported these lasted 4 days	Visits to healthcare professionals had an emotional impact on parents: overwhelmed with decisions, grief & guilt watching their child in pain, struggling to communicate "slowly deteriorate & die" Time & attention required to care for child limited time for social activities, confined to home, contributed to relationship breakdowns Depression, feelings of fear, guilt, remorse, & hopelessness Impacted decision to have further children & impacted siblings as they struggled to understand the disease	Comprehensive information about psychiatric/behavioral signs & symptoms Increase awareness & knowledge of MLD to reduce misdiagnoses & diagnostic delays Treatment priorities: Preference for a treatment that stops the disease progression or slows progression of disease to improve quality of life Therapy to improve speech or communication & reduce decline in mobility, pain, cognitive ability, communication, respiratory problems or food intake
Needham et al. [55]	Communication, daily living skills, socialization, & motor skills were significantly lower for patients with severe MPS II Receptive, expressive & written communication; personal & community daily living skills; play & leisure time; coping skills; & fine motor skills were significantly lower for patients with severe MPS II Within the severe MPS II group, there was a strong significant & negative correlation between all Vineland domains & age. Thus, for those with severe MPS II, the older the patients are, the lower their adaptive behavior functioning	A more severe presentation of MPS II was correlated with more impact (i.e. lower HRQOL, lower family functioning, lower social, physical functioning, daily living & family relationships) for the parents & families Levels of support & ways to maintain psychological health varied widely for parents Parents reported maintaining employment, staying active, going to church, attending therapy, positive thinking, support from friends & family, social networking, & going out with friends supported their psychological health Fear of the child’s death &/or life expectancy, worry for their child’s pain, future level of functioning, how the patient will cope with having MPS II, & social isolation Greatest worry is what would happen to their children if the parent died before the patient	Support systems to improve social life & methods to maintain psychological health
Krantz et al. [51]	Long processing times & lack of knowledge about the disease Society not developed for progressive disease where worsening of symptoms can happen suddenly Limited support & access to contacts in the health &/or social insurance system Adult healthcare system was not seen as fit for purpose	Recurring losses & grief throughout the course of the disease which began with diagnosis & continued with progressive loss of child's function Difficulties finding time to recharge, less time available outside of the family & a loss of relationships with people who used to be close Negative impact on romantic relationships Side-lined siblings The end stage of the disease with the child completely dependent & with possible psychotic symptoms brings a sense of an additional loss, not only related to their death but also a loss of identity Guilt, frustration, hopelessness Shared resilience adapting to new circumstances using acceptance & adopting new shared beliefs about what is important & meaningful in life	Relief from care assistants, relatives & a short-term stay at a care centre where families of children with disabilities could meet Time to recharge without guilt & spending time without affected child was important to maintain family functioning Organizational processes: need for flexibility & workplace adjustments Parental support groups to share knowledge & experiences was a way to create understand & learn from other parents A result-oriented vision including collaborative problems solving within the families & seeing the disease as a common enemy could help with dealing with the grief of the diagnosis
Porter et al. [52]	Two overarching impact domains cognitive/behavioral/psychological & physical health	Cognitive/behavioral/psychological impact: Child: frustration, anxiety/unhappiness in child Child & family: communication, frustration, hyperactivity Family: relationship & social deficits, impulse control/aggressive behaviors, unsafe behaviours, sleep disturbance/night-time waking Physical health impact: Child: Sleep problems, seizures, feeding & maintaining nutrition Child & family: Pain/headaches (experienced & anticipated), mobility, illness/vulnerability to illness Family: digestive issues & toileting	Pressing treatment needs related to communication & behavior Parents highly valued incremental benefits targeting symptoms, to maintain current skills set & treatments that would slow or stop symptom progression or rate of decline Broad as possible access to clinical trials (without age limitations) & later to approved therapies Emphasis on improving quality of life for the child, parents, & family as their primary goal for new treatments Clinical trials that assess outcomes other than, or in addition to, currently used global cognitive measures of evidence for global improvement in cognition
Grant et al. [56]	Older children with MPS III were more intellectual impaired As children with MPS III got older the frequency of their challenging behaviour decreased, but there was no significant relationship with how difficult parents found these behaviours to manage Children with MPS III had a significantly higher level of intellectual disability than the children with ID	Social resources as their highest protective factor & planned future as their lowest protective factor Parents experienced similar levels of perceived social support & reported feeling significantly more supported by their significant other than by family or friends Acceptance was most frequently used coping strategy & self-blame was least used coping strategy Both groups of parents scored above the clinical cut off for anxiety & depression Mean scores of both groups of parents were higher in all categories than those of mothers of children in oncology services indicating high levels of stress in both groups	Behaviour support packages, with appropriate adaptations to learn behaviour management techniques, coping strategies, how to build, resilience factors, & social support networks Flexible strategies to respond to their child’s current needs & unpredictable behavior patterns rather than focusing on future planning Professional psychological support for families during transition periods to discuss their mixed emotional challenges Referral to appropriate psychological services, together with support from relevant parental support groups, who play an important role in providing social & emotional support to parents who are geographically diverse & do not have access to face-to-face support groups
Somanadhan et al. [49]	Frustration waiting long time for diagnosis & spending significant amount of time navigating bureaucracy for basic needs Future is unknown from prognosis to outcome Unsatisfactory collaboration & communication between healthcare professionals Hospital Vs. Home: preferred to be in their home rather than a hospital, aided routine & structure in their regular life	Devastated by the enormity of the debilitating nature of their child’s rare genetic life-limiting condition & the impact on their child’s life expectancy Process of trying to accept their child’s diagnosis was an emotional struggle, they reacted with upset, devastation, anger & shock Life characterised by uncertainty & ambiguity: *life as a roller coaster & living with MPS as a constant battle* Marital relationships markedly strained & fear of siblings becoming side-lined Fear that comes with increasing evidence of visible clinical features & realisation that the gap would widen as they got older Frustration & anger comparing to other conditions with treatments available – particularly those parents of children approaching end of life Parents wanted to be proactive & commonly used reframing strategies Coping achieved through awareness that there were other children with more severe diagnoses	Collaboration & communication between healthcare professionals, especially during initial diagnostic disclosure Improved specialist knowledge of rare condition among healthcare professionals to avoid delays in referral, diagnosis & disease management/treatment Emotionally supportive communication of diagnosis & coping strategies for ongoing challenges of caring for child with progressive condition that has no cure Streamlined, consistent & equitable access to healthcare resources & supports for their child’s condition. E.g., more responsive physiotherapy, occupational therapy, psychology, & social support services Frequent & planned reassessment of children & parents psychosocial & mental healthcare needs
Kuratsubo et al. [60]	Correlation between low social status & psychological problems among parents Many of the parents were seeking psychological counseling	High scores on the BDI-II, suggesting mothers & fathers were in a depressive state. Depression in mothers was serious as compared with previous reports According to the GHQ60 11/21 mothers were considered to be in a state of neurosis 4/16 fathers & 8/21 mothers showed high levels of anxiety Younger mothers with young patients with fewer years since diagnosis tended to be more depressed	Tailored support services, including psychological counseling, home nursing care, supports in workplace & community Early intervention for mental health problems should be provided for younger mothers with few years since the child’s diagnosis
Varvogli et al. [61]	Children had significant developmental & neurological difficulties Progressive worsening of neurological functioning Over half patients had 4 + special needs. The most common were dependence on a wheelchair, developmental delays, feedings through a gastrointestinal tube & vision problems	56% of the mothers had scores in the pathological range on three or more scales Higher MMPI-2 scores were significantly related to the age of mothers, with younger mothers evidencing greater psychopathology Results from the open-ended interview suggest that the child’s age & the circumstances of the diagnosis did not have an impact on the mother’s personality profile	Psychosocial services tailored to reduce the level of anxiety & stress in mothers
Conjin et al.		Posttraumatic Stress: 22%, (7 mothers & 3 fathers) met the criteria for the diagnosis of PTSD. The prevalence in this study is significantly higher among parents of MPS III patients compared to Dutch general population Anxiety & Depression: Mothers of MPS III patients reported significantly higher mean levels of anxiety & depression, & more frequently clinically relevant anxiety & depression than mothers in the reference group Fathers reported more frequently clinically relevant depression compared to fathers in the reference group	Structural monitoring of the psychosocial functioning of MPS III parents Access to professional psychological & peer support from those in the community
Dermer et al. [57]		The average total stress percentile significantly higher than the median normative sample Twenty percent of parents had high levels of stress (> 85th percentile), & 11% had clinically significant levels of stress (> 90th percentile) No significant difference was found between mother & father’s mean total stress score Mothers’ total stress percentile was significantly higher if their child had behavior difficulties	Tailored parent psychological counseling & support early after the initial diagnosis Supportive treatments of children with behavioral difficulties
Ammann-Schnell et al. [62]	Time to diagnosis & number of clinicians families visited in attempt to get diagnosis from first onset of symptoms was very distressing Disease-specific knowledge in parents was high: 71% received disease specific information from clinicians, 51% from other affected families, 46% internet, 14% specific literature Empathic handling of diagnosis, honesty about the severity & comprehensible explanation of the condition was helpful Disturbing factors were insensitive behaviour towards parents, ignorance of the disease from doctors’ part & passing on of unconfirmed diagnosis	MLD families reported significantly poorer HRQOL than the families with healthy children (Total Impact Score, Parent HRQOL & Family Functioning Summary Scores) Significantly poorer Family Functioning observed in the late-infantile MLD families compared to the juvenile MLD families No significant correlations between the severity of the symptoms on the family’s quality of life Mothers spent an average of 88 h caring for child, more often than fathers missed out on or sacrificed a professional career to be able to care for the child at home The diagnostic phase/time to diagnosis & the terminal disease stage represents an especially high burden on families The heavy demands on care, & minimal feedback from the child at that stage are possible reasons for this	Increasing general awareness, about rare diseases including (MLD) among healthcare professionals Encouraging healthcare professionals to listen to parents who report that they are worried about developmental & behavioral features of their children, they have not known before A reliable system of easily accessible diagnostic counseling for healthcare professionals & families, which should involve academic centers to promote rapid diagnosis of rare diseases
Bose et al. [48]	Parents of younger children relieved upon receiving diagnosis vs resignation & numbness in older bereaved parents	Range of emotions identified by frequency (overwhelmed, stressed, anxious, exhausted most frequent) & extensiveness (“gut-wrenching”, “devastating”, anger at healthcare professionals & healthcare system – not invested in learning more about rare diseases/lack of coordination) Stressors; interactions with healthcare professionals / managing multiple needs – feeling of failure, impact on social relationships, highlighting importance of social networks, perceived lack of support contributed to resentment & isolation Confusion deep sadness & uncertainty for the overall progressive nature of disease/ present & anticipated negative impact on child’s QOL Regret & guilt for time lost spent on unaffected siblings Coping (cognitive & behavioural strategies employed to address these emotional experiences) redefining expectations, acceptance of difference in parent experiences to help cope with challenges & limitations of the disease, advocating for child’s needs, becoming involved in promoting disease awareness Collaborative, transparent & consistent healthcare community fosters trust & respect which positively impacted parents	Simple, targeted approaches focusing on improved communication between healthcare professionals & parents Resources for emotional support & counseling to support redefining parent expectations & to promote acceptance Social support system & healthcare community (e.g. advocacy organisations to enhance knowledge & ability to address child’s needs effectively) crucial element in managing overall emotional experience
Killian et al. [63]	Increasing child & parent age associated with decreased parent PCS & increased MCS Parents of children who were older at diagnosis showed poorer parent PCS & improved MCS Greater time period beyond age of regression was associated with poorer parent PCS & improved MCS More severe disease associated with poorer PCS but improved MCS Feeding problems & seizure severity adversely impacted both PCS & MCS	Multivariate predictors: a longer interval beyond regression was associated with poorer PCS Parents who felt limited in the amount of time for their own needs also had poorer PCS, as did parents who sensed tension or conflict at home For parent MCS, increased child’s age was associated with an improved score Feeding problems were associated with poorer MCS. Finally, CHQ items associated with personal worry, lack of time & poor family dynamics described a relationship between increasing severity & poorer MCS For Rett syndrome, clinical severity, as measured by the CSS, associated with poorer PCS but with improved MCS	Access to appropriate psychosocial resources to reduce parent emotional distress, worry & to improve family dynamics Time allocated to cater for parents own needs, especially among parents of children with severe clinical features (child feeding problems & seizure severity) to improve parent quality of life Supports for child feeding problems & seizure severity
Rozensztrauch et al. [54]	75% of children aged 1–2 years experienced a significant regression in acquired skills, with developmental deterioration, severe dementia, with loss of speech, autistic features, & stereotypies Malnutrition (78% of children), epilepsy (43%), impaired mobility (40%), scoliosis (13%), respiratory problems (87%), hyperventilation 22% Average age when symptoms were first noticed was 1 year Child’s average age at diagnosis was 3.5	Malnutrition & feeding difficulties significantly affect family functioning in family relationships dimension Parents were not satisfied with the effects of rehabilitation Children living in urban areas had better access to healthcare professionals than those living in towns & villages	Access to specialists & rehab centres offering early diagnostic services, especially for families living in rural areas Psychological support for parents, especially in early diagnosis stage Assistance with feeding difficulties to improve family relationships Identification of child’s need & targeted assistance with individual problems Healthcare professionals’ help with making appropriate decisions for patient care
Lamb et al. [64]		Parents who shared more of the caregiving responsibilities with other family members had more effective family functioning Less effective functioning was associated with having more than one child with RS & having children who were older when symptoms of RS emerged (more years asymptomatic) 3 key variables were significantly correlated with family functioning: parental self-efficacy, problem-focused coping & emotion focused coping 4 key variables were significantly correlated with adaptation: parental self-efficacy, problem-focused & emotion focused coping, & family functioning Mediation: family functioning is a significant partial mediator of the relationships between adaptation & three key variables: parental self-efficacy, problem-focused coping & emotion focused coping	Interventions to strengthen parent competence in their role to enhance parent adaptation, to increase parental self-efficacy, employing appropriate coping strategies, & facilitating greater family functioning Parent education about importance of family dynamics, to facilitate effective communication about roles & responsibilities, & to encourage other members of the family to provide support for the primary caregiver Referral pathways to support groups & other resources to promote adaptation
Palacios-Ceña et al. [27]	Genetic diagnosis experienced as a pilgrimage: looking for answers, seeking diagnosis: as confirmation of their suspicions & fears as early as possible to avoid delaying treatment Information seeking from specialist healthcare professionals & internet Non-pharmacological therapies (speech therapy, physio) necessary basic pillars for parents to fight illness on another front as they attempt to decrease the physical & cognitive disability of the child	Symptoms: manifold, arising at irregular intervals, leading to a state of alert in the caregivers, which affected their initial feelings of joy after giving birth Challenges managing day-to-day life: integrating treatments & care into daily life & family routine Appointments short with limited number of sessions, difficult & expensive to access continuous therapies in public system Learning to care; with ever-changing problems without a fixed pattern (nutritional problems, bowel problems, sleeping disorders, respiratory disorders, epileptic seizures & stereotypical movements) Sleep, stress & anxiety impact the whole family & parent who experience distress that they are unable to care for their child High impact on family economic resources, direct & indirect indispensable costs associated with hospital care, work readjustments resulting in decreased economic income. In addition, cost of sanitary supplies is considered abusive; no regulations	Economic support & resources for direct & indirect indispensable caregiving costs Equitable access to genetic testing & diagnoses across geographical / residential locations Social support networks to loan or buy second-hand materials
Mori et al. [65]		Living in a remote area, the child being a teenager at baseline, frequent sleep disturbances or behavioural problems, & the type of MECP2 gene mutation were each associated with later poorer parental physical well-being Being a single parent or on a low income was also associated with later poorer physical well-being Child being enterally fed was associated with later poorer emotional well-being Both the physical & emotional well-being of the parent improved when the child was living in out-of-home care	Healthcare professionals being alert to the possibility & need for management of a child’s sleep or emotional disturbance Additional support for parents as child moves through adolescence into early adulthood, including additional physical supports (hoists, modified vehicle) Respite for coping with care demands & facilitating parents’ social life Additional supports for single parents & families showing poor functioning

### Parents' experiences searching for a diagnosis and navigating health and social care systems

#### Battling for a diagnosis

Parents spent an extended length of time and energy visiting numerous clinicians and undergoing multiple diagnostic tests after the birth of their child in the hope of obtaining a diagnosis which was highly emotionally and physically taxing [50, 51]. Extensive challenges connecting with specialist clinical teams who could engage empathetically and listen to parents’ concerns regarding their child’s developmental and behavioral features, that medical teams have not previously considered as part of the diagnostic formulation were identified in several studies [51, 53, 56, 62]. The prolonged delay in achieving a diagnosis incurred substantial delays in accessing timely therapies and was a source of heightened chronic stress which parents reported was like ‘*watching a ticking time bomb’* [49]. As their child’s condition progressed, acute-onset and non-specific disease-related symptomatology, such as severe seizures had a significant traumatic impact on parents’ emotional wellbeing [26, 49, 53, 59, 67, 68] leading to a state of prolonged state of alertness in parents [53].

#### Perpetuating obstacles to management and supports for progressive symptomology

Beyond the diagnostic period, parents experienced ongoing decisional uncertainty and limited access to information about how to manage the range of their child’s non-specific and uncertain neurocognitive and behavioral disease symptoms, which “*arose at irregular intervals*” [50, 52, 53, 65]. Parents reported perpetuating obstacles managing their child’s daily care, without access to first-line non-pharmacological and behavioral therapies (e.g. speech therapy, physiotherapy) to reduce the acceleration of their child’s developmental impairments and cognitive decline [47, 53, 54, 58, 63]. In several studies, parents emphasized that the prolonged delays they experienced in accessing healthcare services combined with the perceived amplification of their child’s progressive physical symptoms and neurocognitive regression compounded the intensity of their distress [47, 51]. Parents experienced frustration, and despair, “*battling*” beurocracy [49] for basic healthcare supports including a medical card or a wheelchair for their child [49, 51, 52, 65]. Profound developmental regression, life-threatening seizures, respiratory complications, motor impairment, sleep disturbances, and associated psychotic manifestations were repeatedly identified as conferring a high physical burden on parents [47, 50, 53, 55, 58, 63]. In addition, uncooperative behaviors including aggression, hyperactivity, agitation, orality, and a loss of communication skills were emotionally distressing for parents, who frequently reported a deficit of available guidance and support to manage and cope with their child’s challenging behaviors [58].

Parents emphasized that healthcare and social insurance systems did not cater for a rare disease where life-threatening symptoms could suddenly develop and progressively cause loss of function [48, 51, 53]. The perceived manageability of their child’s comorbidities and physical disabilities became more difficult with their child’s increasing age [52, 55] and parents described feeling powerless, watching their child being “*eaten away*” by disease [49]. Although some parents had a team of engaged clinicians to provide holistic support for their child in the paediatric healthcare system [51, 62], transitioning to the adult healthcare setting, where clinicians lacked knowledge about their child’s condition and treated co-occurring developmental and behavioral features in isolation caused parents significant additional anguish [48, 51]. For example, parents described experiencing feeling “*trapped in a box*” [49] without support when they no longer belonged in the paediatric healthcare system and had not completed passage to the adult healthcare system [49].

The advanced disease stage had a significant negative impact on parents’ quality of life due to the high level of impairment their child was suffering, the physicality of heavy caregiving demands, coupled with minimal feedback from their child [62]. Challenging symptoms in the advanced stages included blindness, complete loss of mobility, communication and basic sensorimotor function, and independent eating capacity or the requirement for a percutaneous endoscopic gastrostomy tube [62]. Complete dependence on parents, coupled with severe emotional and behavioral impairments including psychotic symptoms brought additional loss and resignation towards the end stage of the disease [48]. While some parents preferred the option to care for their child in the comfort of their own home (e.g. rather than a hospital setting) [49], longitudinal studies indicated parent quality of life improved when the child was living in out-of-home care and when parents were allocated time to cater for their own needs [51, 55, 65].

### The psychosocial impacts of childhood dementia throughout the child’s life and ongoing grief

#### Multiple layers of recurring losses

Parents described an all-consuming experience of chronic sorrow associated with their child’s deteriorating function and progressive clinical features which commonly began after the birth of their child [53], and continued throughout the disease course [51]. As their child matured, physical disabilities including loss of function and mobility had profound “*destroying impact on the child’s body*” and caused their child significant frustration [49]. Parents in turn experienced reciprocal distress, anticipatory grief and intense prolonged emotional heartache watching their child feel anxious, unhappy, and in pain [49, 50, 52]. Further, their child’s marked loss of communication skills impaired parents’ capacity to understand their child’s verbal/non-verbal messages and was perceived as losing a crucial aspect of the parent–child relationship [52]. For example, parents reported their child’s loss of communication skills hindered primary parenting goals and caused parents to question their self-efficacy to meet their child’s evolving healthcare needs [49, 52]. Parents reported a perceived pressure to protect both their child and family from the stigmatization of a “*cruel society*”[49], particularly when their child’s uncooperative behavior (e.g. aggression, irritability, hyperactivity) was negatively misinterpreted by others [51, 58].

Parents described feeling that their life was “*on pause*”, with reduced capacity for social activities outside the family home, whilst they simultaneously grieved the losses of their relationship with their child and their relationships with people who used to be close [50, 51]. Parents also reported intense fear and chronic traumatic distress experiencing their child “*slowly deteriorate and die*” [50] and coinciding communication difficulties with clinical teams who often could not offer appropriate emotional counselling support [51]. Notably, the family and clinical team seeing the disease as a common enemy helped parents to cope with the grief of their child’s diagnosis [51]. The relief from care assistants and a short-term stay at a care centre where families of children with disabilities meet were described as a salvation which provided a source of recovery for parents [51]. Furthermore, parents who accessed psychosocial supports and shared responsibilities with other family members reported enhanced self-efficacy and more effective family functioning [48, 49, 54, 62–65]. On the other hand, the lack of psychosocial supports contributed to resentment and anger [48] especially among parents of children approaching end of life [49]. Parents whose child had died reported experiencing a loss not only related to their child’s death but also a loss of identity [51].

#### Personal costs and sacrifices

Parents reported challenges meeting their physical, social and psychological needs due to the significant demands of their child’s extensive healthcare needs and complex care and management requirements [48, 51, 52]. Parents frequently reported interruption to their professional career to be able to take care of their child, resulting in decreased economic income for the family [53, 62]. The intensity of caregiving demands was illuminated in a study indicating that mothers spent, on average, 88 h per week caring for their child and managing their child’s high-risk behaviors [62]. Additionally, the high direct economic costs of therapies that were indispensable for their child’s care and the indirect costs associated with frequent hospital appointments and work readjustments reinforced economic struggles for parents [53]. Multivariate analyses indicated that having two or more siblings, or being a single parent was associated with poor family functioning and poor physical wellbeing among parents over time [65]. In several studies, parents also reported guilt and tension regarding not being present for the healthy siblings of their ill child [49–51, 65].

#### Quality of life and mental health impacts

Six studies investigated the relationship between a spectrum of disease-specific factors, including the severity of the child’s physical, behavioral and neurocognitive functioning [57–59, 63, 65, 69] with parent quality of life outcomes [57, 59, 69]. Although the heterogeneity of individual dementia phenotypes in the included studies varied both within and across patient conditions, overall, the severity of the child’s diagnostic presentation appeared to have consistent, adverse impacts on parents’ quality of life and mental health outcomes. Poorer quality of life and mental health outcomes were highlighted in studies which compared parents of children with a dementia with other illness conditions (e.g. parents of oncology patients) [56], reference group populations (e.g., healthy controls or norm values) [57, 59], as well as those that investigated subgroups of parents of children with mild and severe forms of the same diagnosis (e.g. Mucopolysaccharidosis II; MPS II)[55, 58]. The severity of the child’s clinical presentation (e.g., degree of developmental regression) was associated with greater adverse impacts on parent quality of life outcomes (e.g., physical health component scores and mental health component scores) [63, 65], lower scores on psychological examinations [60, 61] and lower family functioning [52, 55, 62, 64, 65]. For example, a more severe presentation of MPS II was correlated with lower parent health-related quality of life and lower family functioning in terms of family relationships, communication, stress, decision-making, psychosocial functioning, and psychological status [55].

Several studies indicated clinically-relevant levels of parent stress [57], depression [50, 60], anxiety [56] and post-traumatic stress disorder [56, 59], particularly among younger mothers of recently diagnosed children [60, 61]. Conversely, greater time period beyond their child’s age of regression was associated with improved parent psychological wellbeing [63]. Sleep and mood disturbances contributed heightened emotional and behavioral challenges in children and were associated with poorer parent psychological wellbeing and poorer parent physical wellbeing over time [65]. For example, sleep disturbances and behavioral difficulties translated to exacerbated parent distress, anxiety, sleep quality and subsequent capacity to care for their child’s healthcare needs [51–53, 58, 65]. Additionally, the relationship between the severity of the child’s clinical presentation and parent psychological status [57, 59] was examined in quantitative and qualitative studies which indicated significantly higher stress among parents of children with severe behavioral comorbidities, including aggression and feeding difficulties [54, 58]. Affirming this, qualitative interviews investigating parent psychosocial functioning indicated that the severity and chronicity of their child’s symptom progression conferred high psychological distress [50], and adversely impacted on parents’ health related quality of life [62].

#### Expressions of resilience

Interestingly, although disease severity was shown to predict parents’ psychological status [55], coping and stress reduction strategies, including accessing psychological counselling and social support networks served to alleviate parents’ levels of stress and improved family functioning, regardless of the severity of their child’s condition [49, 51, 52, 58, 64]. In these studies, parents shared that their experience with their child’s condition encouraged them to see life from a new perspective and to adopt new beliefs about what was meaningful in life [51, 52]. To maintain a positive outlook, parents prioritized social connectedness and accessible community supports to facilitate shared learning and emotional support amidst the ongoing challenges [51, 58, 60]. In addition, parents reported that spending time without the affected child was important to maintain balance in the family and to support relationship dynamics between parents and healthy siblings, as well as the couple's relationship [51]. Additionally, quantitative studies investigating family functioning found better quality of life outcomes, including improved psychological wellbeing, among family members who allocated time spent without their affected child to fulfil their personal and family’s needs [63, 65]. Likewise, factors related to family functioning and parent adaptation to their child’s diagnosis found more effective family functioning was associated with greater parent self-efficacy, and enhanced problem and emotion-focused coping [64]. In this study, family functioning was a significant partial mediator of the relationships between adaptation, parent self-efficacy and coping [64].

### Parents’ information and support needs

#### Proactive communication and collective learning

Insufficient information to navigate the slow and arduous diagnostic process was a prevailing theme [49, 50, 58, 62]. Parents advocated for proactive explanations about what tests their child was undergoing and honest discussions to help parents to set realistic expectations regarding what to expect following diagnostic investigations [48]. Accordingly, when honest and comprehensible conversations about their child’s anticipated disease severity were initiated early, this had a significant positive impact on parents’ psychological wellbeing [49, 62] and their capacity to emotionally prepare to care for their child [51]. As with the information gap, parents widely reported a perceived lack of knowledge and openness to learning among clinicians with respect to their child’s condition and subsequent challenges accessing coordinated therapeutic supports for their child’s progressive disease symptomology [53]. Parents emphasized the importance of having clinicians listen to their concerns about the onset of developmental and behavioral symptoms in their child, which clinicians may not have encountered before [62]. Parents underscored that collaborative communication and collective learning between multidisciplinary and sub-specialist clinicians and families was crucial to expediate delayed wait times and barriers to access to therapeutic interventions to halt the acceleration of their child’s cognitive regression, and progressive comorbidities [48, 51, 58, 62].

#### Intense need for urgent therapeutic development

Parents prioritized the urgent need for therapeutic interventions that target their child’s cognitive regression, and behavioral and psychiatric comorbidities as their primary goal for new treatments [50, 52]. For example, parents reported that therapeutic interventions that reduce the pace of their child’s global regression including slowing the rate of decline in cognition, communication and mobility, as conferring the greatest benefit in terms of their quality of life outcomes [50, 52]. Worthwhile approaches to manage challenging behavior included play and distraction techniques, environmental changes and repeated specific reassurance, rather than traditional operant conditioning [58]. To accelerate therapeutic developments, parents emphasized the need for faster pathways to a diagnosis, as well as broader access to clinical trials and approved therapies [52]. To better meet the needs of families, parents advocated for clinical trials to incorporate measures that holistically quantify the impact of their child’s progressive clinical phenotype (e.g., improvements in mobility or treatments for pain management would be meaningful, even in the absence of evidence for improvement in cognition) [51, 52, 66]. Parents of children who were older or had further progressed in their disease course expressed disappointment in the age limitations for past and ongoing clinical trials [52]. Parents proposed that a dedicated space, such as a centre of expertise for rare disease, would optimize clinical practice as it would expand clinician knowledge and facilitate collaboration between clinicians, healthcare providers, patients, and families, [49, 52].

#### Advocacy for meaningful resources and services

To achieve equitable healthcare services to address their child’s basic needs, parents underscored a pressing need for collective advocacy to generate awareness and recognition for the psychosocial support needs of children with dementia, and their parents in hospital and community settings [47, 48, 54, 60, 62]. Parents considered that stage-specific psychoeducational resources and support during different stages of their child’s condition trajectory was critical to support emotional adaption to their child’s progressive condition [51, 56, 57, 60]. For example, parents advocated for tailored psychoeducation about how to identify and manage complex psychiatric and behavioral symptoms that their child could develop, and how to integrate their child’s “*ever-changing*” and progressive care requirements into daily life and family routine [53]. Parents strongly recommended family counselling in a private space to provide an opportunity to collectively learn about their child’s condition [51, 62] and to help strengthen interfamilial functioning surrounding the diagnostic period [48, 58]. Studies demonstrated psychological counselling was especially important for younger mothers with few years since the child’s diagnosis [60]. Similarly, parents shared that opportunities to emotionally connect with others who understood their circumstances and did not hold disparaging views of their child’s uncooperative behaviors was necessary to provide parents with emotional support and reassurance that they were advocating appropriately for their child [51–53]. In terms of additional psychosocial resources, parents considered that in-home nursing and respite care, flexible supports in the workplace, and accessible community resources were necessary to reduce and treat parent psychological sequelae [47, 48, 54, 55, 60, 62].

## Discussion

**Fig. 3:**
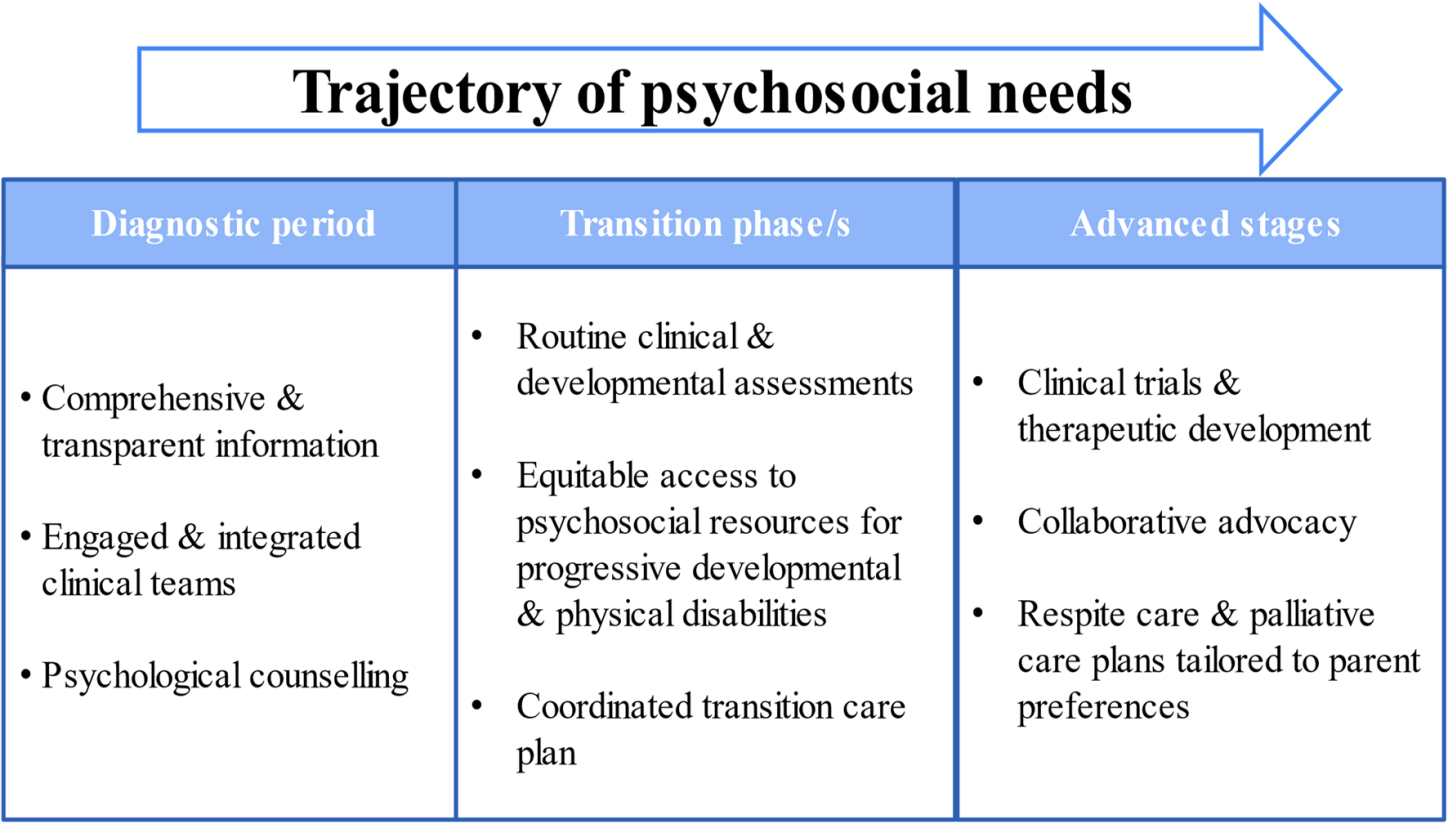
Trajectory of psychosocial needs

Despite emerging knowledge of individually rare childhood dementia conditions, the collective psychosocial impacts of childhood dementias remain under-explored. This is the first systematic review to comprehensively integrate current international evidence documenting the healthcare experiences, psychosocial impacts and unmet information and support needs shared by parents of children with dementia. We identified 3 key overarching themes from 19 studies (Fig. 2). Firstly, parents experience a range of challenges navigating quality health and social care for their child owing to the rare, life-limiting, and progressive nature of their child’s dementia condition [49, 52, 58]. Difficulties accessing clinical teams equipped to provide early and coordinated care for their child’s complex progressive condition, compounded by a deficit of condition-specific information, impart a high physical and mental toll on parents [53, 56]. Secondly, psychosocial challenges are manifold and encompass physical, economic, social, emotional and psychological implications, which frequently precipitate prolonged grief and impaired quality of life among parents [54, 62, 63, 65]. Thirdly, our review identified priority areas that can be targeted with future interventions to protect and promote parents’ psychosocial wellbeing during key stages along the trajectory of their child’s condition (Fig. 3). These priority areas include providing additional evidence-based and integrated psychosocial support to parents during; (1) their child’s diagnostic phase; (2) periods of transition between different healthcare or community care settings; and (3) the advanced stages of the disease, when children lose key skills, such as language or mobility [56, 67].

Our first objective was to investigate parents’ healthcare experiences managing their child’s condition. The most prevalent obstacle that presented across the studies was achieving a diagnosis for their child and connecting with clinical teams who could provide honest and comprehensive information and counselling support about the uncertainty and potential severity of their child’s condition [62]. Persistent barriers accessing information and therapeutic interventions to reduce the acceleration of their child’s progressive neurocognitive decline and deteriorating function cultivated significant grief and frustration among parents, who felt powerless without a clinical team to support them [49]. Our findings extend on emerging rare disease research highlighting that early engagement with coordinated healthcare teams that are equipped to communicate appropriately with families during the “diagnostic odyssey” and to advocate for the child’s progressive healthcare needs, may have a substantial positive impact on parents’ psychosocial wellbeing, and their capacity to care for their child [49, 51, 62, 70]. Importantly, our review identified that the cumulative uncertainties and chronic traumatic stress endured by parents are exacerbated by concurrent progressive global deteriorating function [49, 51, 52] and the fear of their child’s imminent death [48, 51, 55]. Global clinical guidelines highlight the importance of adopting a trauma-informed approaches to care and support families affected by progressive and severely life-limiting disease, including providing timely referrals to paediatric palliative care services [71, 72]. Earlier access to paediatric palliative care services with can improve a child and family’s quality of life through the provision of holistic care and gently introducing conversations about end of life throughout the various phases of a child’s condition [73, 74]. Our findings support that providing early and appropriate integrated care, considering the specific needs of the families and involving both generalist and subspecialist paediatric palliative care teams may improve parents’ preparedness for end of life [51, 53, 56, 58].

A second key finding was that complex childhood dementia phenotypes had wide-ranging psychosocial impacts on parents’ physical, emotional, social, psychological and economic wellbeing [51–54, 60]. Analysis revealed multiple domains of unmet need that impact child and parent quality of life, including neurocognitive regression, loss of communication skills, downstream cognitive-behavioral challenges, sleep disturbances, uncooperative behaviors, as well as progressive physical impairments including pain and mobility [52]. Our findings extend on global research demonstrating that rare diseases and childhood dementias impose significant economic costs far surpassing some of the costliest chronic diseases, to individual households and the broader health systems [75, 76]. Regarding psychological impacts, often, the early age of onset, and the rate of insidious decline in their child’s neurocognitive capacity hastens the demand on parent emotional and psychological adaption, resulting in intensifying psychological isolation and sequalae [77, 78]. Interestingly, although disease severity was shown to predict parents’ psychological wellbeing [55], expressions of parent resilience associated with adaptive coping strategies were highlighted across several studies, [49, 51, 58], regardless of the severity of their child’s condition [52, 64]. Our findings draw parallels with burgeoning research emphasizing the importance of accessible psychosocial resources to promote coping as more reliable predictors for parent quality of life than disease-specific factors [79, 80]. Additionally, our results lend support to national guidelines recommending the application of individually tailored applied behavioural analysis therapy as an intervention to support managing the neurobehavioral aspects of certain childhood dementia conditions [72]. Our finding that parents' psychosocial needs progress during distinct stages of their child’s condition trajectory align with broader conceptual frameworks, which attest that early and integrated healthcare and psychosocial supports are required to support parents’ evolving needs throughout the child’s lifetime [81]. Given the extensive and wide-ranging demands associated with parenting a child with dementia elicited through this research synthesis, integrated family-centred and trauma-informed psychosocial resources tailored to leverage parents’ adaptive coping skills are likely to be important empowerment tools for parents to alleviate psychological and social isolation [78, 82].

Importantly, while unprecedented progress has been achieved in understanding the cellular pathogenesis of many individually rare childhood dementia causing conditions, efficacious treatments are unavailable for the majority [83]. Currently, there are over 70 genetic disorders known to cause childhood dementia [16] and many of these conditions are detected late, adding to the high level of suffering endured by patients and their families [62]. For parents of children with rare diseases, clinical trials are often their only hope to benefit from the pipeline of therapeutic developments [84] and our review emphasized parents’ pressing desire for as broad as possible access to clinical trials and to subsequently approved therapies [52]. Further, our results highlight paediatric clinical trials targeting reduction in the rate of decline of clinical features (e.g. deteriorating motor function and mobility) offers a source of intervention that can complement pharmaceutical approaches and improve parent quality of life outcomes [52]. Collaboration and knowledge sharing achieved through rare disease initiatives has been shown to improve the quality of patient care and is of benefit to researchers, clinicians, and families alike, in considering appropriate referral pathways for available clinical trials and therapeutic interventions [85, 86]. Establishing an integrated and interdisciplinary center of expertise with a wide periphery of clinician involvement may be a strategic approach to assist parents and clinicians in several ways. First, it would facilitate information flow to circumvent the obstacles parents and clinicians face in accessing information, and clinical trials [87]. Secondly, the center could prove advantageous for connecting families to appropriate community-based services, to alleviate social and psychological isolation and to empower families to cope with the uncertainty of their child’s rare and progressive condition [48]. Thirdly, a centralized and interdisciplinary center of expertise engaging informed decision-makers, parents, and clinician researchers would provide a sustainable platform that is conductive to social influence to drive awareness and advocacy to address the shared unmet needs of children and families [88, 89].

### Strengths and limitations

While the findings of this review provide valuable information to accelerate the development of enhanced healthcare and psychosocial resources for families, there are limitations. First, the review contained only articles published in English and hence, additional studies might have been overlooked. Although the review included studies from multiple countries, the experiences and needs of parents in low- and middle-income countries; and those from remote regions, lower socioeconomic and culturally and linguistically diverse populations were underrepresented [90]. Research to understand how we can develop and provide healthcare services for families in low- and middle-income countries is crucial to develop culturally appropriate resources which are aligned to their specific needs [91]. Equally, other priority populations include those from remote regions, lower socioeconomic background and that are Indigenous or otherwise culturally and linguistically diverse [92].

This research was carried out by a cross-disciplinary team whose members contributed a broad and diverse knowledge base. A potential limitation of the current systematic search related to the variable and inconsistent use of terminology to define different conditions that are identified as childhood dementias. While the criteria for defining the childhood dementia diagnoses was not always clearly delineated in specific studies, the authors agreed on set inclusion criteria, as delineated in published literature prior to the data extraction process [1, 11]. Four authors engaged in decisions about inclusion/exclusion in the review, and articles were critically appraised by two authors to ensure methodological rigour and to avoid risk of bias. Additionally, the wide range of study quality and the use of different instruments to assess quality of life outcomes in the individual studies should be considered when interpreting the quantitative results. Two studies directly compared the quality of life of parents of children with a dementia with another illness conditions [56, 62] and two studies relied on population-based norms to describe parent quality of life and psychological wellbeing [57, 59]. Thirdly, few studies explicitly linked their research to a theoretical framework and only two studies reported sample size estimations and power analyses [55, 57]. Importantly, the use of validated instruments within a theory-driven framework may be a useful future approach to facilitate the development of psychosocial resources for parents that will be applicable across their child’s lifespan [93].

### Future directions

As scientific understanding of childhood dementia continues to evolve, an increasing number of families will receive this diagnosis for their child. With this knowledge, streamlined access to multidisciplinary and interdisciplinary healthcare teams, including subspeciality paediatric palliative care teams should be prioritised to provide holistic healthcare and psychosocial support for children and their families [94]. While our review focussed on the impact of caring for a child with dementia from the perspective of parents, several studies acknowledged that unaffected siblings may be adversely impacted by the increased time and energy required to care for the sick child [48–51, 55]. Future research expanding on the present research is necessary to investigate the psychosocial impacts of childhood dementia conditions on siblings and to improve the depth of understanding regarding what psychosocial resources may be required to support other family members in the community [55]. This is particularly important given that siblings are often tasked with caring for their brother or sister from a young age and the physical and emotional responsibilities can become more prevalent over time as the parents age and are less able to undertake all the necessary caring responsibilities [95, 96].

Secondly, we acknowledge that clinicians can also be impacted by the professional and emotional demands of caring for children with severe and rare neurodegenerative conditions and their families [97–100]. Clinicians experience a protracted clinical care remit, navigating pervasive clinical uncertainty associated with genetic discovery and prognostication challenges, in the face of limited natural history data, and limited hopes for targeted therapeutics across time [101]. The increased complexity of health care delivery for this group of severe and complex neurodevelopmental and neurodegenerative conditions has elevated the importance of understanding the clinician experience, and for continued research to understand the natural history of childhood dementia disorders to enable clinicians to better support families [12]. Engagement with paediatric palliative care services specialised in caring for children with life-limiting conditions and their families ensures a layer of holistic consultancy support [102, 103]. Our results highlight that the need for clinician education and training in compassionate communication models about the progression of childhood dementia, as well as guidance about navigating appropriate psychosocial supports for families. Future research assessing the perceptions and psychosocial support needs of clinicians and parents collectively may help to map out what resources can be co-developed to empower clinicians to optimally care for patients and their families [104].

## Conclusion

Parents’ health and social care experiences are characterised their child’s rare, life-limiting and progressively impairing childhood dementia specific complexities. Our review demonstrated that parents share a tranche of overlapping challenges, especially related to the psychiatric and behavioral impacts of their child’s cognitive regression and the perpetuating psychosocial challenges that result. Nonetheless, many parents employed adaptive coping strategies and demonstrated significant resilience. Findings highlight the need for early access to coordinated healthcare services and trauma-informed integrated psychosocial supports to alleviate parent psychological distress. The unifying focus on the collective lived experiences of parents underscores the urgent need to increase awareness of the impact of childhood dementias among all stakeholders, to drive investment, integrated services, and interventional research.

### Supplementary Information

**Additional file 1**: **Table S1** Database search terms. **Tables S2** and **S3** Quality analysis summary scores for qualitative articles.

## Data Availability

The author confirms that all data analysed and generated during this study are included in this published article. Furthermore, the secondary sources and data supporting the findings of this study were all publicly available at the time of submission. One can obtain the relevant materials from the references below.
