# Supplementary material for: The psychosocial impact of childhood dementia on children and their parents: a systematic review
Source: Orphanet J Rare Dis. 2023 Sep 7;18:277. doi: 10.1186/s13023-023-02859-3 (PMC10486052; doi:10.1186/s13023-023-02859-3)
Supplement: Supplementary file 1 — Additional file 1: Table S1 Database search terms. Tables S2 and S3 Quality analysis summary scores for qualitative articles. [file 13023_2023_2859_MOESM1_ESM.docx]

**Supplementary materials**

**Supplementary Table 1**. Database search terms.

| **Population** | AND | **Illness** | AND | **Outcome of Interest** |
| --- | --- | --- | --- | --- |
| Parents |  | "Childhood Dementia" OR "Mitochondrial disorders" OR "Mitochondrial disease" OR "Rett Syndrome" OR "Zellweger Spectrum" OR "Zellweger Syndrome" OR "Menkes Disease” OR Mucopolysaccharidosis type III" OR “Sanfilippo syndrome” OR "Glycine Encephalopathy" OR "Vanishing White Matter Disease" OR "X-linked Adrenoleukodystrophy" OR “Cerebral adrenoleukodystrophy” OR "NCL" OR ''Batten Disease" OR "Canavan disease" OR "Cobalamin C Disease" OR "MPS I" OR "Hurler Syndrome" |  | Psychosocial care |
| OR |  | OR |  | OR |
| Caregiver |  | “Niemann-Pick disease” OR  “MPS II” OR “Hunter Syndrome” OR “Metachromatic Leukodystrophy” OR  “Krabbe” OR “Tay Sachs” OR “Sandhoff disease” |  | Quality of life |
| OR |  | OR |  | OR |
| Family |  | disorders of mitochondrial function"/ OR leigh syndrome / or mitochondrial encephalopathy |  | Psychosocial intervention |
| OR |  | OR |  | OR |
| Child |  | disorders of mitochondrial function"/ OR leigh syndrome / or mitochondrial encephalopathy |  | Health service needs and demands |
|  |  |  |  | OR |
|  |  |  |  | Mental health |
|  |  |  |  | OR |
|  |  |  |  | Psychological well-being |
|  |  |  |  | OR |
|  |  |  |  | Coping behavio*r |

**Supplementary Table 2 & 3**

Quality analysis summary scores for qualitative articles

| **Reference** |  | 1. Question objective sufficiently described? | 2. Study design evident & appropriate? | 3. Context for the study clear? | 4. Connection to a theoretical framework/wider body of knowledge? | 5. Sampling strategy described, relevant & justified? | 6. Data collection methods clearly described & systematic? | 7. Data analysis clearly described & systematic? | 8. Use of verification procedures to establish credibility? | 9. Conclusions supported by the results? | 10. reflexivity of the account? | TOTAL SUM | TOTAL POSSIBLE SUM | SUMMARY SCORE |
| --- | --- | --- | --- | --- | --- | --- | --- | --- | --- | --- | --- | --- | --- | --- |
| Bose et al., | Yes | 1 | 1 | 1 | 1 | 1 | 1 | 1 | 1 | 1 | 1 | 20 | 20 | 1 |
|  | Partial |  |  |  |  |  |  |  |  |  |  | 0 |  |  |
| Somanadhan et al., | Yes | 1 | 1 | 1 | 1 | 1 | 1 | 1 |  | 1 |  | 16 | 20 | 0.9 |
|  | Partial |  |  |  |  |  |  |  | 1 |  | 1 | 2 |  |  |
| Yazdani et al., | Yes | 1 | 1 | 1 | 1 | 1 | 1 | 1 | 1 | 1 | 1 | 20 | 20 | 1 |
|  | Partial |  |  |  |  |  |  |  |  |  |  | 0 |  |  |
| Eichler et al., | Yes | 1 | 1 | 1 | 1 | 1 | 1 | 1 |  | 1 |  | 16 | 20 | 0.9 |
|  | Partial |  |  |  |  |  |  |  | 1 |  | 1 | 2 |  |  |
| Krantz et al., | Yes | 1 | 1 | 1 | 1 |  | 1 | 1 | 1 | 1 |  | 16 | 20 | 0.9 |
|  | Partial |  |  |  |  | 1 |  |  |  |  | 1 | 2 |  |  |
| Porter et al., | Yes | 1 | 1 | 1 | 1 | 1 | 1 | 1 | 1 | 1 |  | 18 | 20 | 0.95 |
|  | Partial |  |  |  |  |  |  |  |  |  | 1 | 1 |  |  |
| Palacios-Ceña et al., | Yes | 1 | 1 | 1 | 1 | 1 | 1 | 1 | 1 | 1 |  | 18 | 20 | 0.95 |
|  | Partial |  |  |  |  |  |  |  |  |  | 1 | 1 |  |  |

Quality analysis summary scores for quantitative articles

| **Reference** |  | 1. Question objective sufficiently described? | 2. Study design evident & appropriate? | 3. Method of subject/ comparison group or source of information/input variables described & appropriate? | 4.Subject (& comparison group, if applicable) characteristics sufficiently described? | 5. If interventional & r&om allocation possible, was it described? | 6. If interventional & blinding of investigators possible was it reported? | 7. If interventional & blinding of participants was possible, was it reported? | 8. Outcome & (if applicable) exposure measures well defined & robust to measurement/misclassification, 53-55? Means of assessment reported? | 9. Sample size appropriate? | 10. analytic measures decribed/justified & reported? | 11. Some estimate of variance is reported for the main result? | 12. Controlled for confounding? | 13. results reported in sufficient detail? | 14. Conclusions reported for the results? | TOTAL SUM | TOTAL POSSIBLE SUM | SUMMARY SCORE |
| --- | --- | --- | --- | --- | --- | --- | --- | --- | --- | --- | --- | --- | --- | --- | --- | --- | --- | --- |
| Rozensztrauch et al., | Yes | 1 | 1 | 1 | 1 |  |  |  | 1 |  | 1 | 1 |  | 1 |  | 16 | 20 | 0.9 |
|  | Partial |  |  |  |  |  |  |  |  | 1 |  |  |  |  | 1 | 2 |  |  |
|  | N/A |  |  |  |  | 1 | 1 | 1 |  |  |  |  | 1 |  |  | 4 |  |  |
|  | No |  |  |  |  |  |  |  |  |  |  |  |  |  |  |  |  |  |
| Needham et al., | Yes | 1 | 1 | 1 | 1 |  |  |  | 1 | 1 | 1 | 1 |  | 1 | 1 | 20 | 22 | 0.954545 |
|  | Partial |  |  |  |  |  |  |  |  |  |  |  | 1 |  |  | 1 |  |  |
|  | N/A |  |  |  |  | 1 | 1 | 1 |  |  |  |  |  |  |  | 3 |  |  |
|  | No |  |  |  |  |  |  |  |  |  |  |  |  |  |  |  |  |  |
| Grant et al., | Yes | 1 | 1 | 1 | 1 |  |  |  | 1 |  | 1 | 1 |  | 1 | 1 | 18 | 20 | 0.95 |
|  | Partial |  |  |  |  |  |  |  |  | 1 |  |  |  |  |  | 1 |  |  |
|  | N/A |  |  |  |  | 1 | 1 | 1 |  |  |  |  | 1 |  |  | 4 |  |  |
|  | No |  |  |  |  |  |  |  |  |  |  |  |  |  |  |  |  |  |
| Dermer et al., | Yes | 1 | 1 | 1 | 1 |  |  |  | 1 | 1 | 1 | 1 |  | 1 | 1 | 20 | 20 | 1 |
|  | Partial |  |  |  |  |  |  |  |  |  |  |  |  |  |  | 0 |  |  |
|  | N/A |  |  |  |  | 1 | 1 | 1 |  |  |  |  | 1 |  |  | 4 |  |  |
|  | No |  |  |  |  |  |  |  |  |  |  |  |  |  |  |  |  |  |
| Hoffmann et al., | Yes | 1 | 1 | 1 | 1 |  |  |  | 1 |  | 1 | 1 |  | 1 | 1 | 18 | 20 | 0.95 |
|  | Partial |  |  |  |  |  |  |  |  | 1 |  |  |  |  |  | 1 |  |  |
|  | N/A |  |  |  |  | 1 | 1 | 1 |  |  |  |  | 1 |  |  | 4 |  |  |
|  | No |  |  |  |  |  |  |  |  |  |  |  |  |  |  |  |  |  |
| Conjin et al., | Yes | 1 | 1 | 1 | 1 |  |  |  | 1 | 1 | 1 | 1 |  | 1 | 1 | 20 | 20 | 1 |
|  | Partial |  |  |  |  |  |  |  |  |  |  |  |  |  |  | 0 |  |  |
|  | N/A |  |  |  |  | 1 | 1 | 1 |  |  |  |  | 1 |  |  | 4 |  |  |
|  | No |  |  |  |  |  |  |  |  |  |  |  |  |  |  |  |  |  |
| Kuratsubo et al., | Yes | 1 |  |  |  |  |  |  | 1 |  | 1 |  |  | 1 |  | 8 | 20 | 0.7 |
|  | Partial |  | 1 | 1 | 1 |  |  |  |  | 1 |  | 1 |  |  | 1 | 6 |  |  |
|  | N/A |  |  |  |  | 1 | 1 | 1 |  |  |  |  | 1 |  |  | 4 |  |  |
|  | No |  |  |  |  |  |  |  |  |  |  |  |  |  |  |  |  |  |
| Varvogli et al., | Yes | 1 |  |  |  |  |  |  |  |  |  |  |  |  |  | 2 | 18 | 0.555556 |
|  | Partial |  | 1 | 1 | 1 |  |  |  | 1 |  | 1 | 1 |  | 1 | 1 | 8 |  |  |
|  | N/A |  |  |  |  | 1 | 1 | 1 |  | 1 |  |  | 1 |  |  | 5 |  |  |
|  | No |  |  |  |  |  |  |  |  |  |  |  |  |  |  |  |  |  |
| Ammann‑Schnell et al., | Yes | 1 | 1 | 1 | 1 |  |  |  | 1 | 1 | 1 | 1 |  | 1 | 1 | 20 | 22 | 0.954545 |
|  | Partial |  |  |  |  |  |  |  |  |  |  |  | 1 |  |  | 1 |  |  |
|  | N/A |  |  |  |  | 1 | 1 | 1 |  |  |  |  |  |  |  | 3 |  |  |
|  | No |  |  |  |  |  |  |  |  |  |  |  |  |  |  |  |  |  |
| Killian et al., | Yes | 1 | 1 | 1 | 1 |  |  |  | 1 | 1 | 1 | 1 |  | 1 | 1 | 20 | 22 | 0.954545 |
|  | Partial |  |  |  |  |  |  |  |  |  |  |  | 1 |  |  | 1 |  |  |
|  | N/A |  |  |  |  | 1 | 1 | 1 |  |  |  |  |  |  |  | 3 |  |  |
|  | No |  |  |  |  |  |  |  |  |  |  |  |  |  |  |  |  |  |
| Lamb et al., | Yes | 1 | 1 | 1 | 1 |  |  |  | 1 | 1 | 1 | 1 |  | 1 | 1 | 20 | 20 | 1 |
|  | Partial |  |  |  |  |  |  |  |  |  |  |  |  |  |  | 0 |  |  |
|  | N/A |  |  |  |  | 1 | 1 | 1 |  |  |  |  | 1 |  |  | 4 |  |  |
|  | No |  |  |  |  |  |  |  |  |  |  |  |  |  |  |  |  |  |
| Mori et al., | Yes | 1 | 1 | 1 | 1 |  |  |  | 1 | 1 | 1 | 1 | 1 | 1 | 1 | 22 | 22 | 1 |
|  | Partial |  |  |  |  |  |  |  |  |  |  |  |  |  |  | 0 |  |  |
|  | N/A |  |  |  |  | 1 | 1 | 1 |  |  |  |  |  |  |  | 3 |  |  |
|  | No |  |  |  |  |  |  |  |  |  |  |  |  |  |  |  |  |  |
